# Eight-Year Trends in the Effect of the Great East Japan Earthquake on Obstetrics Outcomes: A Study from the Fukushima Health Management Survey

**DOI:** 10.3390/life13081702

**Published:** 2023-08-08

**Authors:** Hyo Kyozuka, Tetsuya Ohhira, Tsuyoshi Murata, Shun Yasuda, Kayoko Ishii, Seiji Yasumura, Keiya Fujimori, Hitoshi Ohto, Kenji Kamiya

**Affiliations:** 1 Department of Obstetrics and Gynecology, School of Medicine, Fukushima Medical University, Fukushima 960-1295, Japan; 2 Radiation Medical Science Center for the Fukushima Health Management Survey, Fukushima 960-1295, Japan; 3 Department of Epidemiology, School of Medicine, Fukushima Medical University, Fukushima 960-1295, Japan; 4 Department of Public Health, School of Medicine, Fukushima Medical University, Fukushima 960-1295, Japan

**Keywords:** delivery, natural disaster, obstetric outcomes, cesarean section, environmental disaster

## Abstract

Information regarding the longitudinal effects of natural/environmental disasters on obstetrics outcomes is limited. This study aimed to analyze the longitudinal changes in obstetrics outcomes over 8 years after the Great East Japan Earthquake and the Fukushima power plant accident. We used data from the first 8 years of the Pregnancy and Birth Survey by the Fukushima prefectural government, launched in 2011. We compared data on obstetrics outcomes by year and divided Fukushima Prefecture into six districts based on administrative districts. Longitudinal changes in the occurrence of preterm birth before 37 gestational weeks, low birth weight, and anomalies in newborns were accessed using the Mantel–Haenszel test for trends in all six districts. Overall, 57,537 participants were included. In 8 years, maternal age, conception rate after sterility treatment, and cesarean section delivery incidence increased. Although significant differences were observed in preterm birth and low birth weight occurrence among districts, there was no significant trend in the occurrence of preterm birth, low birth weight, and anomalies in newborns in all six districts of Fukushima Prefecture. The Great East Japan Earthquake and Fukushima power plant accident were associated with increased cesarean section delivery incidence but had no significant adverse effects on obstetrics outcomes.

## 1. Introduction

**Figure 1:**
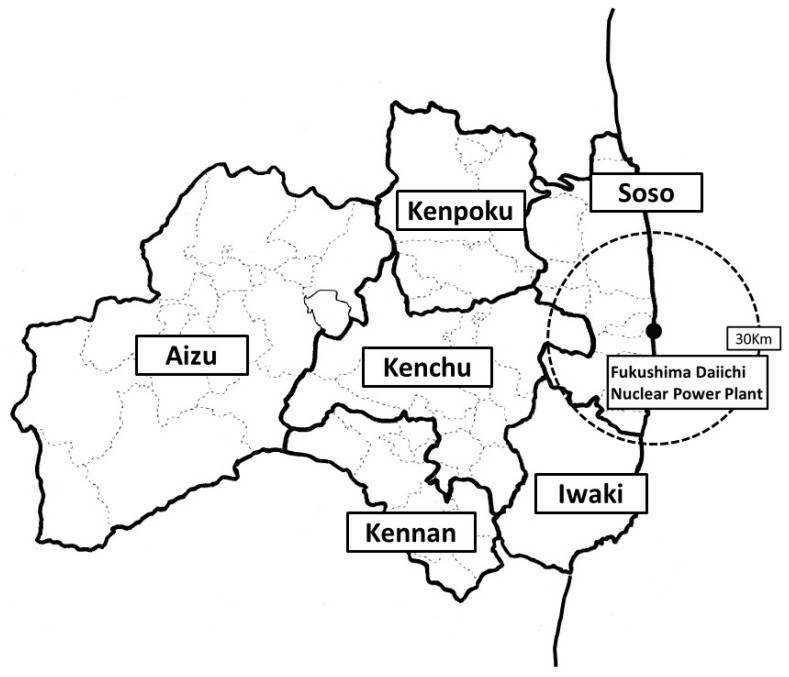
Geographic information of Fukushima Prefecture. Geographic districts were first classified into seven areas, Kenpoku, Kenchu, Kennan, Soso, Iwaki, Aizu, and Minami-Aizu, and, then, the Aizu and Minami-Aizu areas were combined and called the Aizu area. The black dot within the Soso district represents the location of the Fukushima Daiichi Nuclear Power Plant. The circular dotted line indicates a distance of 30 km from the Fukushima Daiichi Nuclear Power Plant.

The Great East Japan Earthquake, which occurred on 11 March 2011, along with the subsequent tsunami and nuclear accident at the Fukushima Daiichi Nuclear Power Plant, was the most devastating event in recent Japanese history. In the Fukushima Prefecture, thousands of deaths were reported due to the tsunami, with the devastation affecting many people living in the coastal areas of the Soso and Iwaki districts. After the power plant accident, residents living in coastal areas were forced to evacuate suddenly. The Aizu district, located in a mountainous region in Fukushima Prefecture and far from the power plant, experienced less damage than the coastal regions (Figure 1).

After the disaster, the Fukushima Health Management Survey (FHMS), a population-based study that included geographical and birth information used to evaluate pregnancy outcomes, was launched by Fukushima Prefecture to provide valuable data on the investigation of the health effect of low radiation dose and disaster-related stress in 2011 [1]. It is well established that disasters can significantly impact perinatal outcomes. Numerous studies have reported associations between such disasters and various aspects of perinatal health [2]. Even without direct exposure to the disaster, the surrounding circumstances can increase the risk of adverse pregnancy outcomes, including more unplanned pregnancies and sexually transmitted infections [2]. Specifically, regarding the Great East Japan Earthquake, some studies have reported its impact on perinatal outcomes in 2011 [3,4,5,6,7]. It has been shown that this disaster impacted both immediate outcomes and longer-term postnatal factors, such as the incidence of postnatal depression and the state of breastfeeding nutrition. Only a few studies have examined chronological trends in the occurrence of perinatal outcomes after the disaster. The chronological trends in pregnancy outcomes after the Great East Japan Earthquake is of worldwide interest; currently, the FHMS maintains the data from the investigation of the effect of this disaster on pregnancy and infant care.

This study aimed to examine the 8-year chronological trends in perinatal outcomes after the Great East Japan Earthquake in Fukushima Prefecture by district, using data from the FHMS.

## 2. Materials and Methods

### 2.1. Study Design

We used the maternal survey questionnaire results from the FHMS. The methods used for the FHMS and maternal survey have been previously reported [1]. The maternal survey is a population-based study conducted as part of the FHMS launched by the Fukushima Prefecture government in 2011 to assess the health condition of pregnant women and their neonates after the Great East Japan Earthquake. In this study, Fukushima Prefecture was divided into six districts [3], and women who received the maternal and child health handbook since 1 August 2010, were included. The maternal and child health handbook is a unique perinatal healthcare initiative in Japan. The handbooks help maintain a record of women’s antenatal and postnatal checkups by physicians. The self-report questionnaire was mailed to the participants on 18 January 2012. The mothers were asked to refer to their maternal and child health handbooks while completing the questionnaire. The issuance of the maternal and child health handbooks is carried out through administrative services. The linkage between the issuance of these handbooks and the mailing addresses was carried out through the administration. The response to the questionnaire was made through postage-paid return mail. This study was approved by the local ethics review committee of the authors’ institution (Approval No. 1317), and written informed consent was obtained from all participants.

The participants were pregnant women and their newborns delivered between 11 March 2011 and 31 December 2018. Cases of delivery before 11 March 2011, women who received a maternal and child health handbook outside Fukushima Prefecture, those with insufficient data, women pregnant at the time of the survey, women who had had an abortion, and mothers of triplets were excluded from this analysis. 

### 2.2. Maternal Information and Obstetrics Outcome

The self-report questionnaire included maternal information such as the geographic district where women received the maternal and child health handbook when pregnant, year of delivery, maternal age at delivery, single or multiple gestational pregnancies, gestational weeks at delivery, mode of pregnancy, and mode of delivery, and neonatal information such as neonatal birth weight, sex of newborn, and presence of anomalies in newborns. Geographic districts were classified into six areas, namely, Kenpoku, Kenchu, Kennan, Soso, Iwaki, and Aizu (Figure 1). Delivery before 37 gestational weeks was defined as preterm birth (PTB), and a birth weight of <2500 g was defined as low birth weight (LBW). The mode of pregnancy was categorized as a natural pregnancy or the use of sterility treatment, such as ovulation induction, artificial insemination, or in vitro fertilization. The mode of delivery was categorized into vaginal delivery or cesarean section. Major anomalies reported in newborns were as follows: cataract, cardiac malformation, kidney or urinary tract malformation, spina bifida, microcephaly, hydrocephalus, cleft lip or palate, intestinal atresia (esophagus, duodenum, and ileum), imperforate anus, and poly- or syndactylism. Every anomaly reported in the questionnaire was defined as a major anomaly.

### 2.3. Statistical Analysis

The maternal characteristics of neonates were categorized into six groups according to birth year. The Chi-square test was used to compare the categorical variables, such as response rate and perinatal outcomes among areas per year. The extended Mantel–Haenszel Chi-square test for linear trends was used to analyze the trends in proportion between 2011 and 2018. The Jonckheere–Terpstra trend test was used to analyze trends in continuous variables between 2011 and 2018. SPSS ver. 26 (IBM Japan, Tokyo) was used for data analysis. A *p*-value of <0.05 was considered statistically significant.

## 3. Results

**Figure 2:**
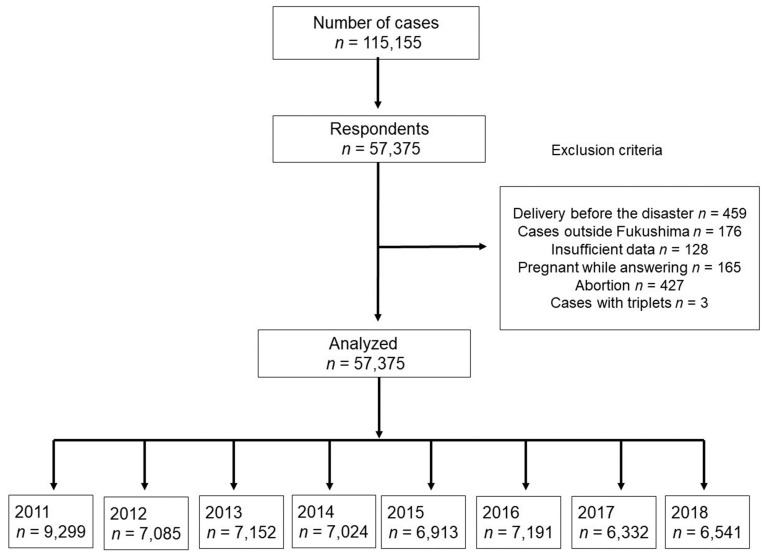
Study flow.

The survey questionnaire was mailed to 115,155 women who had experienced pregnancy during the study period. In total, 57,537 women (response rate 50.0%) responded to the questionnaire. Women who had given birth before 11 March 2011 (*n* = 459), who received maternal and child health handbooks outside Fukushima Prefecture (*n* = 176), for whom sufficient data were unavailable (*n* = 128), who were pregnant at the time of the survey (*n* = 165), who had had an abortion (*n* = 427), and those with triplets (*n* = 3) were excluded from the study. After applying these exclusion criteria, 57,375 women were finally included in the analyses. The number of cases in each of the eight years from 2011 to 2018 inclusive was 9299, 7085, 7152, 7024, 6913, 7191, 6332, and 6541, respectively (Figure 2).

Table 1 shows the chronological changes in response rates by district. The Mantel–Haenzel test showed a significantly decreasing trend in the response rate from 58.2% in 2011 to 51.4% in 2018 (*p* < 0.001). This was observed in all districts except Kennan (*p* = 0.652). The Chi-square test showed differences in response rates among districts. During the study period, Kenpoku had the highest response rate, except in 2011, when the area with the highest response rate was Soso (65.6%), the location of the coastal area and the Fukushima Daiichi power plant. 

Table 2 summarizes the characteristics of the respondents based on the year of delivery. Although maternal age, gestational age at delivery, and ratio of male newborns showed significant differences during the study period (*p* < 0.001, *p* < 0.001, and *p* = 0.046, respectively), there was no significant difference in mean neonatal weight (*p* = 0.912). Additionally, there were no significant differences in the rate of single pregnancy (*p* = 0.368). The conception rate after sterility treatment and cesarean delivery tended to increase (*p* < 0.001 and *p* = 0.005, respectively).

Table 3 shows the chronological change in the occurrence of preterm birth by district. The Chi-square test showed no significant difference in the occurrence of preterm birth among the districts, except in 2012 (*p* = 0.020). The overall rate of preterm birth was 4.6% in 2011 and increased to 5.2% by 2018. However, there was no significant trend in the occurrence of preterm birth between 2011 and 2018 in all areas (*p* = 0.197). 

Table 4 shows the chronological change in the occurrence of LBW by district. There was no significant difference in the occurrence of LBW during the study period. In 2011, a significant difference in the occurrence of LBW was observed between Kempoku (7.6%) and Iwaki districts (10.3%). In 2012, there was also a significant difference with respect to the occurrence of LBW between Kenpoku (7.6%) and Aizu districts (11.3%). The overall rate of LBW was 8.6% in 2011 and rose to 9.1% by 2018; however, no significant increasing trend was observed over time (*p* = 0.500).

Table 5 shows the chronological change in the occurrence of anomalies in newborns. The rate of anomalies across all regions was 2.85% in 2011; however, it was 2.23% in 2018. There was no significant trend in the occurrence of newborn anomalies between 2011 and 2018 in all areas (*p* = 0.069), and the Kenchu district showed a decrease in the rate of anomalies in newborns (*p* = 0.011).

Table 6 delineates the incidence rate of anomalies each year, classified into 11 different types. Excluding the category of “Others,” the most frequently observed anomaly each year was “Cardiac malformation,” which was found at a rate of 1.01% in 2013. There was no significant trend in the occurrence of each neonatal anomaly.

## 4. Discussion

To our knowledge, this is the first population-based study that examined chronological trends for pregnancy outcomes following the disaster. Although the response rate in 2011 was the highest, especially in the Soso district, where the most disaster-related damage had occurred, the response rate decreased over the years, with more than 50% reported in 2018. We also found an increasing trend for mean maternal age, rate of conception after sterility treatment, and rate of cesarean delivery over the years. Regarding PTB, LBW, and fetal anomalies, there were no distinct changes in the trend of occurrence. 

The response rate in this study was approximately 50%, which varied significantly over the years and between districts. While Kenpoku constantly had a higher response rate than the remaining districts, Soso had a higher rate only in 2011. This difference may be related to the concern of pregnant women in the Kenpoku and Soso districts that they were exposed to relatively higher radiation doses. Disasters potentially influence a range of reproductive outcomes [8]. Previous studies examined the effects of exposure to disasters on pregnancy outcomes, and these exposures were usually from so-called “attacks” such as the World Trade Center Disaster, the bombing attack in Serbia, and the Madrid train bombing; environmental and chemical disasters such as the Bhopal gas release in India, the Three Mile Island accident, and the Chernobyl accident; and natural disasters such as earthquakes, hurricanes, floods, and avalanches [2]. The Great East Japan Earthquake and Fukushima Daiichi nuclear accident form a complex disaster because they included natural disasters such as the Great Earthquake and tsunami and environmental/industrial disasters such as the nuclear power plant accident. 

### 4.1. Disaster in Fukushima and Preterm Birth or LBW

Findings on the association between environmental/chemical disasters and gestational age or birth weight are conflicting. For instance, Goldman et al. reported that the Love Canal disaster in the USA showed no significant association with gestational age among 227 residents [9]. Meanwhile, Levi et al. reported that the Chernobyl accident affected gestational age and maternal anxiety among 88 Swedish women who were early in their pregnancies during the disaster [10]. Inconsistent with our study, the Wenchuan earthquake disaster in China increased the risk of PTB. Tan et al. compared the incidence of PTB between 6638 pregnant women before the Wenchuan earthquake disaster and 6365 pregnant women after the disaster [11]. The incidence of PTB was 5.6% and 7.4%, respectively, significantly higher after the disaster (*p* < 0.01). In Japan, high-risk pregnancies have increased due to advanced maternal age and complicated pregnancies [12,13,14]. The incidences of PTB at <37 gestational weeks (5.7%) and LBW of <2500 g (9.4%) in the 6 areas in the study have almost been stable after the disaster. The evidence on the effect of the Fukushima disaster is also conflicting. Hyashi et al. used the same FMHS data and found that the Great East Japan Earthquake had no significant association with the incidence of PTB at <37 gestational weeks during the first year of the disaster among total Fukushima residents [15]. Several pregnant women were forced to evacuate during the disaster, resulting in maternal depressive symptoms [4,16]. Suzuki et al. reported that pregnant women who changed their perinatal checkup institution due to medical indication were significantly associated with shorter gestational duration (β = −10.6, *p* < 0.001) and preterm birth (adjusted odds ratio, 8.5; 95% confidence interval, 5.8–12.5) compared with women who visited only one institution [17]. 

The association between the environment or a natural disaster and fetal growth is also controversial [15,18,19,20,21]. Regarding the Great East Japan Earthquake and Fukushima Daiichi nuclear accident, we have previously reported no evidence that the disaster increased the incidence of small gestational age in the Fukushima Prefecture during the first year of the disaster [5]. Using an institution-based investigation of the coastal area where the most catastrophic damage occurred, Leppold et al. reported no increased proportions in preterm births or LBW in any year after the disaster (merged post-disaster risk ratio of preterm birth: 0.68, 95% confidence interval: 0.38–1.21 and LBW birth: 0.98, 95% confidence interval: 0.64–1.51) [22]. In Japan, pregnant women may have better access to relief programs or receive adequate support from their families, society, and government during disasters [4].

### 4.2. Congenital Anomalies

The association between disasters and congenital anomalies is a major public concern. Several major environmental or industrial disasters have been related to congenital anomalies. These include the nuclear reactor accidents at Chernobyl, Ukraine, in 1986 and Three Mile Island, Pennsylvania, in 1979. The accident at Chernobyl involved a much larger radiation dose exposure and affected more people than the Three Mile Island or Fukushima incidents. Reviews on the effect of the Chernobyl disaster indicated increased microcephaly and neural tube defects [23,24,25]. However, the incidence of most congenital anomalies did not increase in most European countries [26,27,28]. Previous studies have reported that 2–3% of all newborns have a major congenital abnormality, which is detectable at birth [29,30] In Japan, from 2011 to 2016, the incidence of congenital anomalies was 2.43–2.59%, according to a report of the International Clearinghouse for Birth Defects Surveillance and Research Japan Center [31]. Using a Japanese birth cohort study, which included 12,804 pregnant women in Fukushima Prefecture, Kyozuka et al. reported that the prevalence of major congenital anomalies at delivery between 2011 and 2014 in Fukushima Prefecture was 1.6–3.2%, depending on maternal age [6]. Using the same data set as this study, Fujimori et al. reported that the occurrence of congenital anomalies in Fukushima Prefecture during the first year after the disaster was 2.72% (238/8672) [3]. 

Environmental endocrine disrupters, a group of compounds with potentially adverse health effects, are thought to be associated with cryptorchidism [32]. Kojima et al. suggested that it is difficult to clarify the prevalence of cryptorchidism due to the complexities of the design settings of epidemiological surveys of this disease. They rejected the hypothesis that cryptorchidism increased anywhere in Japan due to the Fukushima Daiichi Nuclear Power Plant accident [33]. Hirata et al., using the All Japanese Cardiovascular Surgery Database, reported no increase in the number of patients with congenital heart disease during the period 2010–2013 [34].

Our study has several strengths. In Japan, few epidemiological studies include pregnant women. Large-scale studies and data supported by the government are considered valuable. In addition, we obtained relatively precise data on gestational ages and birth weights from participants’ maternal and child health handbooks. Nevertheless, this study has some limitations. First, because the response rate was only 50–60% throughout the study period, the incidence of negative outcomes may have been overestimated due to the possible over-representation of women affected the most by the disasters, especially those pregnant between 2011 and 2012. Second, as this study used a self-administered questionnaire, it is assumed that the mothers answered correctly, especially regarding fetal anomalies. Lastly, this survey analyzed each district but did not investigate the relationship with individual radiation exposure doses.

## 5. Conclusions

In conclusion, the findings of our study and previous study suggest that the Great East Japan Earthquake and associated disasters had little effect on exposed pregnant women in the first 8 years following the earthquake. A better understanding of the adverse reproductive effects of disasters is required to allow as much preparedness as is needed during an emergency response to prevent mortality and morbidity [4]. Further studies to determine whether the disaster causes early pregnancy loss, such as miscarriage or abortion, or psychological-associated complications, are warranted.

## Figures and Tables

**Table 1:** Chronological change in response rate by each district.

	2011	2012	2013	2014	2015	2016	2017	2018	*p*-Value ^†^
Response rate									
All of Fukushima, %	58.2	49.1	47.3	46.8	47.8	51.3	47.1	51.4	<0.001
(Res/Send)	(9299/15,972)	(7085/14,420)	(7152/15,108)	(7024/15,017)	(6913/14,454)	(7191/14,019)	(6332/13,435)	(6541/12,730)	
Kenpoku, %	62.8 *	55.5 *	53.2 *	52.4 *	52.3 *	55.9 *	50.9 *	56.5 *	<0.001
(Res/Send)	(2289/3647)	(1857/3347)	(1936/3637)	(1841/3515)	(1806/3453)	(1875/3352)	(1634/3212)	(1702/3015)	
Kenchu, %	59.3	48.7	44.5 *	44.8 *	45.2 *	49.8 **	46.8	51.1	<0.001
(Res/Send)	(2858/4819)	(2067/4243)	(1982/4453)	(1961/4376)	(1924/4261)	(2065/4150)	(1862/3980)	(2006/3923)	
Kennan, %	50.2 *	48.1	48.5	46.5	47.9	51.1	45.1	50.0	0.652
(Res/Send)	(631/1256)	(560/1164)	(588/1213)	(553/1188)	(560/1168)	(571/1118)	(473/1048)	(504/1008)	
Soso, %	65.6 ^†^	43.7 *	45.4	42.2 *	44.2 *	43.6 *	40.5 *	42.3 *	<0.001
(Res/Send)	(963/1468)	(500/1145)	(535/1178)	(512/1213)	(523/1183)	(511/1171)	(442/1091)	(424/1003)	
Iwaki, %	55.9 *	47.8	45.1 **	45.8	46.6	50.1	45.5	49.1 **	<0.001
(Res/Send)	(1515/2711)	(1203/2516)	(1195/2649)	(1213/2648)	(1148/2461)	(1192/2377)	(1054/2317)	(1034/2105)	
Aizu, %	50.4 *	44.8 *	46.3	45.5	49.4	52.8	48.5	52.0	0.001
(Res/Send)	(1043/2071)	(898/2005)	(916/1978)	(944/2077)	(952/1928)	(977/1851)	(867/1787)	(871/1676)	
*p*-value ^‡^	<0.001	<0.001	<0.001	<0.001	<0.001	<0.001	<0.001	<0.001	

^†^ *p*-value was calculated using the Mantel–Haenszel test for trend. ^‡^ *p*-value was calculated using the Chi-square test. * *p* < 0.01, ** *p* < 0.05.

**Table 2:** Characteristics of respondents based on year of delivery.

	2011	2012	2013	2014	2015	2016	2017	2018	*p*-Value
Maternal age, mean ± SD	30.7 ± 5.0	31.0 ± 5.0	31.3 ± 5.0	31.4 ± 5.1	31.6 ± 5.0	31.4 ± 5.0	31.6 ± 5.0	31.8 ± 5.0	<0.001 ^§^
*n*	8598	6940	7044	6940	6815	7091	6288	6463	
Twin pregnancy, %	0.9	0.9	1,1	1.0	0.9	0.8	0.8	0.9	0.368 ^‡^
Gestational week, mean ± SD	38.9 ± 1.7	38.9 ± 1.7	38.9 ± 1.7	38.9 ± 1.8	38.8 ± 1.8	38.9 ± 1.7	38.8 ± 1.7	38.8 ± 1.8	<0.001 ^§^
*n*	8566	6926	7015	6930	6801	7086	6286	6453	
Neonatal weight, mean ± SD	3013 ± 418	2993 ± 435	3003 ± 435	2996 ± 455	2994 ± 445	2999 ± 432	3007 ± 432	3003 ± 438	0.912 ^§^
*n*	8603	6935	7088	6999	6859	7122	6319	6504	
Mode of pregnancy									
Sterility treatment, %	4.3	5.4	6.0	6.6	6.9	7.9	8.0	8.4	<0.001 ^‡^
Sex of newborn									
male, %	51.4	52.5	50.4	51.3	50.1	50.3	50.7	50.8	0.046 ^‡^
Stillbirth, %	0.25	0.29	0.34	0.21	0.25	0.21^†^	0.22 ^†^	0.18 ^†^	0.690 ^‡^
Mode of delivery									
cesarean delivery, %	20.7	21.7	20.3	21.0	22.3	21.2	21.8	22.6	0.005 ^‡^

^†^ excluded confirmed abortion and stillbirth cases, or cases of neonates not confirmed to survive, before sending the questionnaires. ^‡^ *p*-value was calculated using the Mantel–Haenszel test for trend. ^§^ *p*-value was calculated using the Jonckheere–Terpstra trend test.

**Table 3:** Chronological change in the occurrence of preterm birth by each district.

	2011	2012	2013	2014	2015	2016	2017	2018	*p*-Value ^†^
All of Fukushima, *n* (%)	395 (4.6)	393 (5.6)	373 (5.3)	375 (5.4)	386 (5.6)	380 (5.3)	335 (5.3)	341 (5.2)	0.197
Kenpoku, *n* (%)	91 (4.3)	82 (4.5) *	94 (4.9)	96 (5.2)	105 (5.9)	91 (4.9)	72 (4.4)	89 (5.3)	0.247
Kenchu, *n* (%)	114 (4.3)	127 (6.2)	95 (4.8)	107 (5.5)	119 (6.2)	103 (5.0)	103 (5.6)	108 (5.4)	0.254
Kennan, *n* (%)	28 (4.7)	23 (4.1)	33 (5.7)	25 (4.6)	34 (6.1)	30 (5.3)	27 (5.7)	29 (5.8)	0.197
Soso, *n* (%)	38 (4.3)	31 (6.5)	35 (6.6)	31 (6.1)	30 (5.8)	29 (5.7)	21 (4.8)	9 (2.2)	0.207
Iwaki, *n* (%)	71 (5.1)	65 (5.5)	69 (5.9)	72 (5.9)	46 (4.1)	63 (5.3)	62 (5.9)	56 (5.5)	0.886
Aizu, *n* (%)	53 (5.4)	65 (7.3) *	47 (5.2)	44 (4.7)	52 (5.5)	64 (6.6)	50 (5.8)	50 (5.7)	0.962
*p*-value ^‡^	0.633	0.020	0.501	0.718	0.215	0.498	0.481	0.111	

^†^ *p*-value was calculated using the Mantel–Haenszel test for trend. ^‡^ *p*-value was calculated using the Chi-square test. * *p* < 0.05.

**Table 4:** Chronological change in the occurrence of low-birth-weight infants by each district.

	2011	2012	2013	2014	2015	2016	2017	2018	*p*-Value ^†^
All of Fukushima, *n* (%)	736 (8.6)	640 (9.3)	681 (9.6)	683 (9.8)	650 (9.5)	659 (9.3)	584 (9.3)	589 (9.1)	0.500
Kenpoku, *n* (%)	160 (7.6) **	138 (7.6) *	171 (8.9)	168 (9.2)	155 (8.7)	151 (8.1)	155 (9.5)	145 (8.6)	0.107
Kenchu, *n* (%)	217 (8.3)	208 (10.3)	191 (9.7)	192 (9.8)	208 (10.9)	187 (9.2)	166 (9.0)	180 (9.0)	0.908
Kennan, *n* (%)	47 (8.0)	51 (9.2)	58 (9.9)	49 (9.1)	54 (9.8)	56 (9.9)	38 (8.1)	46 (9.3)	0.756
Soso, *n* (%)	70 (7.9)	43 (9.3)	47 (8.9)	57 (11.3)	56 (10.9)	46 (9.1)	50 (11.3)	34 (8.2)	0.254
Iwaki, *n* (%)	143 (10.3) *	101 (8.6)	120 (10.3)	119 (9.8)	99 (8.7)	118 (10.0)	98 (9.3)	99 (9.7)	0.759
Aizu, *n* (%)	99 (10.2)	99 (11.3) **	94 (10.4)	98 (10.4)	78 (8.4)	101 (10.4)	77 (8.9)	85 (9.7)	0.210
*p*-value ^‡^	0.031	0.025	0.761	0.736	0.099	0.349	0.638	0.867	

^†^ *p*-value was calculated using the Mantel–Haenszel test for trend. ^‡^ *p*-value was calculated using the Chi-square test. * *p* < 0.01, ** *p* < 0.05.

**Table 5:** Chronological change in the occurrence of anomalies in newborns by district.

	2011	2012	2013	2014	2015	2016	2017	2018	*p*-Value ^†^
All of Fukushima, *n* (%)	238 (2.85)	161 (2.35)	170 (2.44)	163 (2.36)	150 (2.21)	176 (2.49)	153 (2.44)	144 (2.23)	0.069
Kenpoku, *n* (%)	55 (2.66)	39 (2.17)	44 (2.32)	54 (2.99)	31 (1.75)	41 (2.22)	40 (2.47)	41 (2.45)	0.684
Kenchu, *n* (%)	78 (3.07)	49 (2.43)	50 (2.60)	49 (2.54)	48 (2.54)	57 (2.81)	34 (1.85)	36 (1.82)	0.011
Kennan, *n* (%)	24 (4.20)	13 (2.39)	9 (1.56)	11 (2.04)	18 (3.26)	12 (2.14)	9 (1.92)	10 (2.01)	0.088
Soso, *n* (%)	18 (2.09)	13 (2.86)	9 (1.75)	9 (1.79)	7 (1.36)	13 (2.60)	8 (1.82)	12 (2.88)	0.800
Iwaki, *n* (%)	38 (2.81)	26 (2.23)	35 (3.04)	22 (1.83)	27 (2.41)	26 (2.24)	33 (3.15)	22 (2.15)	0.694
Aizu, *n* (%)	25 (2.63)	21 (2.40)	23 (2.58)	18 (1.93)	19 (2.04)	27 (2.80)	29 (3.36)	23 (2.67)	0.408
*p*-value ^‡^	0.268	0.970	0.411	0.261	0.181	0.787	0.099	0.607	

^†^ *p*-value was calculated using the Mantel–Haenszel test for trend. ^‡^ *p*-value was calculated using the Chi-square test.

**Table 6:** Chronological change in the occurrence of major anomalies in newborns.

	2011	2012	2013	2014	2015	2016	2017	2018	*p*-Value ^†^
All anomalies	238/8345	161/6858	170/6954	163/6915	150/6790	176/7064	153/6278	144/6455	0.069
%	2.85%	2.35%	2.44%	2.36%	2.21%	2.49%	2.44%	2.23%	
Cataract	1	2	1	0	0	1	1	1	0.743
%	0.01%	0.03%	0.01%	0.00%	0.00%	0.01%	0.02%	0.03%	
Cardiac malformation	77	54	70	53	53	66	38	60	0.445
%	0.92%	0.79%	1.01%	0.77%	0.78%	0.93%	0.61%	0.93%	
Kidney/urinary tract malformation	22	14	12	24	14	16	25	14	0.451
%	0.26%	0.20%	0.17%	0.35%	0.21%	0.23%	0.40%	0.22%	
Spina bifida	5	5	3	5	4	3	4	3	0.678
%	0.06%	0.07%	0.04%	0.07%	0.06%	0.04%	0.06%	0.05%	
Microcephaly	1	1	0	1	0	0	0	0	0.129
%	0.01%	0.01%	0.00%	0.01%	0.00%	0.00%	0.00%	0.00%	
Hydrocephalus	1	3	4	2	1	2	1	1	0.478
%	0.01%	0.04%	0.06%	0.03%	0.01%	0.03%	0.02%	0.02%	
Cleft lip/palate	17	14	14	14	14	11	8	7	0.079
%	0.20%	0.20%	0.20%	0.20%	0.21%	0.16%	0.13%	0.11%	
Intestinal atresia	6	7	7	9	4	10	7	5	0.710
%	0.07%	0.10%	0.10%	0.13%	0.06%	0.14%	0.11%	0.08%	
Imperforate anus	4	2	2	5	2	2	3	4	0.695
%	0.05%	0.03%	0.03%	0.07%	0.03%	0.03%	0.05%	0.06%	
Poly/syndactylism	22	15	22	14	14	18	16	13	0.554
%	0.26%	0.22%	0.32%	0.20%	0.21%	0.25%	0.25%	0.20%	
Others	104	61	55	63	58	64	59	54	0.070
%	1.25%	0.89%	0.79%	0.91%	0.85%	0.91%	0.94%	0.84%	

^†^ *p*-value was calculated using the Mantel–Haenszel test for trend.

## Data Availability

The data that support the findings of this study are available from the corresponding author, H.K., upon reasonable request.
